# B-Lymphoid Tyrosine Kinase Crosslinks Redox and Apoptosis Signaling Networks to Promote the Survival of Transplanted Bone Marrow Mesenchymal Stem Cells

**DOI:** 10.34133/research.0660

**Published:** 2025-04-15

**Authors:** Fei Zhang, Tao Wang, Lei Wei, Zhihong Xie, Lijun Wang, Hong Luo, Fanchao Li, Qinglin Kang, Wentao Dong, Jian Zhang, Xuesong Zhu, Chuan Wang, Liang Liang, Wuxun Peng

**Affiliations:** ^1^ Department of Emergency Surgery, The Affiliated Hospital of Guizhou Medical University, Guiyang, Guizhou 550004, China.; ^2^ Laboratory of Emergency Medicine, The Affiliated Hospital of Guizhou Medical University, Guiyang, Guizhou 550004, China.; ^3^ Department of Orthopedics, Warren Alpert Medical School of Brown University,Providence, RI 02912, USA.; ^4^ Department of Critical Care Medicine, West China Hospital of Sichuan University, Chengdu, Sichuan 610041, China.; ^5^ Department of Orthopedics, Shanghai Jiao Tong University Affiliated Sixth People’s Hospital, Shanghai 200233, China.; ^6^ Department of Orthopedics, The First Affiliated Hospital of Soochow University, Suzhou, Jiangsu 215000, China.; ^7^ Department of Orthopedics, Guizhou Provincial People’s Hospital, Guiyang, Guizhou 550002, China.

## Abstract

Stress-induced apoptosis presents an obstacle to bone marrow mesenchymal stem cell (BMSC) transplantation to repair steroid-induced osteonecrosis of the femoral head (SONFH). Thus, appropriate intervention strategies should be explored to mitigate this. In our previous study, we discovered a new subgroup of BMSCs—the oxidative stress-resistant BMSCs (OSR-BMSCs)—which can survive the oxidative stress microenvironment in the osteonecrotic area, through a mechanism that currently remains unclear. In this study, we found that B-lymphoid tyrosine kinase (BLK) may be the crucial factor regulating the oxidative stress resistance of OSR-BMSCs, as it is highly expressed in these cells. Knockdown of BLK eliminated oxidative stress resistance, aggravated oxidative stress-induced apoptosis, reduced the survival of OSR-BMSCs in the oxidative stress microenvironment of the osteonecrotic area, and greatly weakened the transplantation efficacy of OSR-BMSCs for SONFH. By contrast, BLK was weakly expressed in oxidative stress-sensitive BMSCs (OSS-BMSCs). Overexpression of BLK in susceptible OSS-BMSCs allowed them to acquire oxidative stress resistance, inhibited oxidative stress-induced apoptosis, promoted their survival in the osteonecrotic area, and improved the transplantation efficacy of OSS-BMSCs for SONFH. Mechanistically, BLK concurrently activates redox and apoptotic signaling networks through its tyrosine kinase activity, which confers oxidative stress resistance to BMSCs and inhibits their stress-induced apoptosis of BMSCs. Herein, we report that OSR-BMSCs have intrinsic oxidative stress resistance that is conferred and mediated by BLK. This finding provides a potential new intervention strategy for improving the survival of transplanted BMSCs and the therapeutic efficacy of BMSC transplantation for SONFH.

## Introduction

Steroid-induced osteonecrosis of the femoral head (SONFH) is a common and refractory orthopedic disease [1–3]. The collapse rate of the femoral head within 3 years after necrosis is >80%. Because the pathological process after the collapse of the femoral head is difficult to reverse, the disability rate is extremely high, which seriously affects the activities that affected patients can engage in, and their quality of life [4–6]. Therefore, early treatment of SONFH is essential for reducing the risk of collapse of the femoral head and preventing the development of disability in the hip joint [7,8]. Recent preclinical studies have tested stem cell transplantation as a regenerative therapy for osteonecrosis and have reported a number of beneficial results, indicating positive prospects for this therapeutic approach [9–12]. It has been shown that reduction or dysfunction of in situ bone marrow mesenchymal stem cells (BMSCs) in the femoral head is associated with the pathogenesis and subsequent self-repair disorders associated with SONFH [13–16]. Transplantation of exogenous healthy BMSCs for the treatment of SONFH is expected to supplement the stem cell pool with normal osteogenic function, thereby promoting bone repair and regeneration. However, the effects of BMSC transplantation for repairing SONFH are not often as good as expected [17–19]. Recent studies have confirmed that the osteonecrotic area is a microenvironment of oxidative stress [20–23], and our previous results confirmed that the pathogenesis and progression of SONFH are accompanied by oxidative stress (Fig. S1A to K). When we transplanted BMSCs to repair SONFH, many of the transplanted cells underwent stress-induced apoptosis in the osteonecrotic area, and the number of surviving BMSCs was insufficient to compensate for the lost or dysfunctional in situ BMSCs in the femoral head—which limited the ability of the BMSCs to repair bone defects (Fig. S1L to T). Therefore, stress-induced apoptosis currently represents the main preclinical obstacle to the use of BMSC transplantation to repair SONFH, and improving the survival of the transplanted cells is crucial for overcoming this limitation.

The survival of BMSCs under oxidative stress involves the cross-linking regulation of redox and apoptotic signaling networks [24–26]. For example, redox signaling pathways such as extracellular signal-regulated kinases (ERKs), nuclear factor erythroid 2-related factor 2 (Nrf2), and forkhead box O (FOXO) can mediate the expression of intracellular antioxidants, thereby inhibiting or eliminating reactive oxygen species (ROS) and stabilizing intracellular redox homeostasis to alleviate peroxide damage [27–30]. Apoptosis-related signaling pathways such as tumor protein 53 (P53), stress-activated protein kinase (SAPK), protein kinase B (Akt), and nuclear factor-κB (NF-κB) can mediate the expression of apoptosis-related proteins in the cells and block or promote the activation of apoptotic signal cascades to interfere with apoptosis [31–34]. Although some studies have tried to inhibit stress-induced apoptosis by maintaining intracellular redox balance or blocking apoptotic signals, the inhibitory effect of a single regulatory mechanism on stress-induced apoptosis has been limited. Antioxidation involves increasing the expression and activity of antioxidants to eliminate excessive ROS, but it cannot effectively block the activation of apoptotic signal cascades. Anti-apoptosis involves blocking the activation of apoptotic signal cascades by regulating the expression and activity of apoptosis-related proteins; however, it cannot reverse the peroxidative damage caused by ROS accumulation [35–39]. Therefore, to improve the survival of transplanted BMSCs in an oxidative stress microenvironment, redox and apoptotic signaling networks must be co-regulated. In our previous study on the treatment of early-stage SONFH via BMSC transplantation, we found that the transplanted BMSCs showed differential adaptability to oxidative stress—where only a small number were able to adapt to the oxidative stress microenvironment and survive in the osteonecrotic area (Fig. S1). The surviving cells showed resistance to oxidative stress, so they were temporarily named oxidative stress-resistant BMSCs (OSR-BMSCs; Fig. S2). However, the potential mechanism whereby OSR-BMSCs acquire resistance to oxidative stress remains unexplored. If we can find the key factor that synergistically regulates the redox and apoptotic signaling networks in OSR-BMSCs and use its regulatory mechanism to enable transplanted BMSCs to acquire resistance to oxidative stress, we would have an effective method to promote the survival of transplanted BMSCs under oxidative stress conditions and improve the efficacy of their transplantation.

In this study, we identified OSR-BMSC subgroups and then screened the redox and apoptotic signaling networks in them using a combination of quantitative and phosphorylated proteomics. By analyzing the related kinase–substrate interaction networks, we then successfully screened out the crucial factor that synergistically regulates these redox and apoptotic signaling networks: B-lymphoid tyrosine kinase (BLK). On this basis, this study further revealed the role and mechanism of BLK in terms of regulating BMSCs to acquire resistance to oxidative stress and the role of BLK resistance to stress-induced apoptosis in BMSCs that have been transplanted to repair SONFH.

## Results

### BLK is associated with the oxidative stress resistance of OSR-BMSCs

**Fig. 1.**
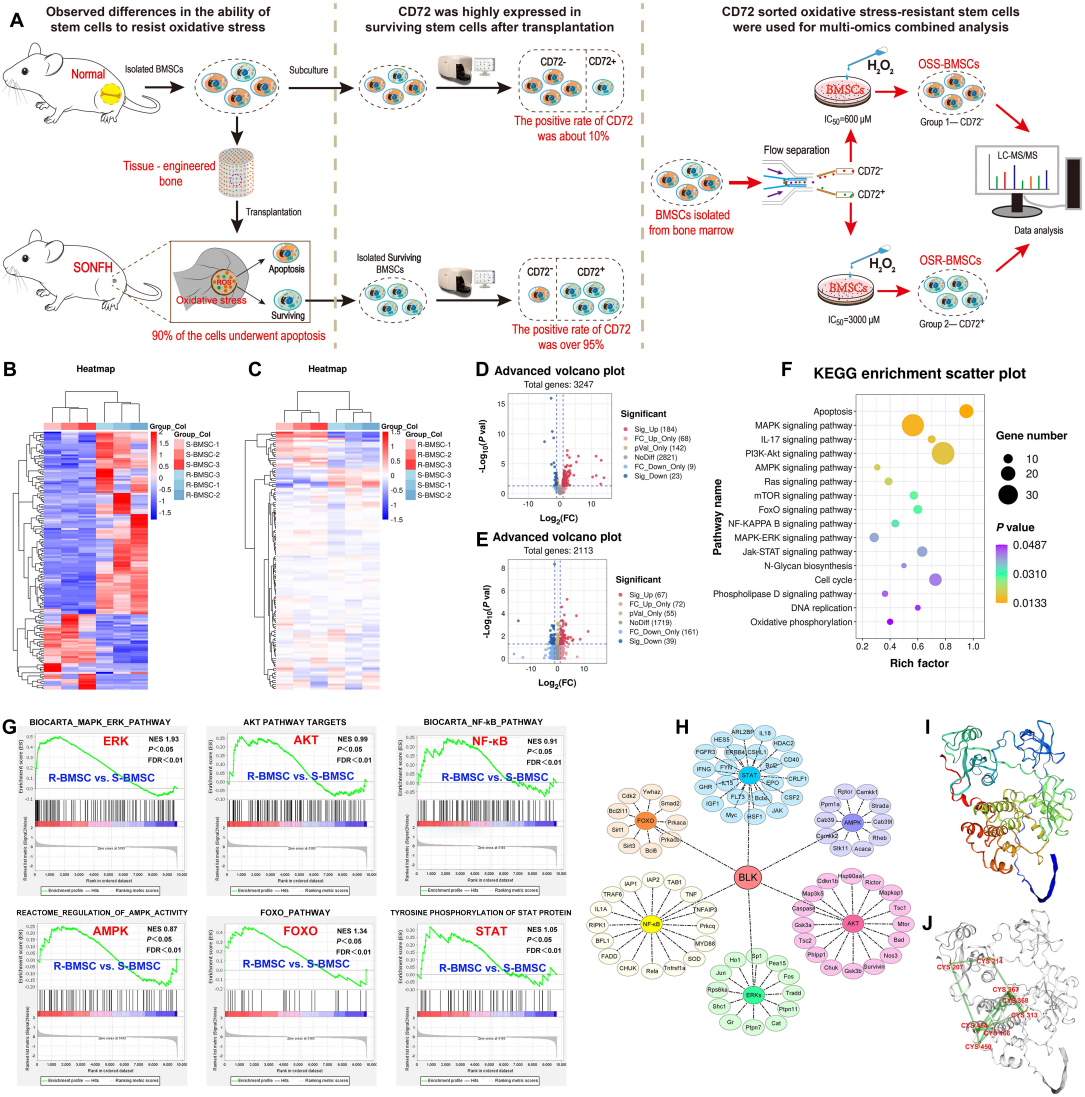
BLK is associated with the oxidative stress resistance of OSR-BMSCs. The crucial factor, BLK, that synergically regulates the redox and apoptotic signaling networks was screened from OSR-BMSCs using proteomics and phosphorylated proteomics. (A) Schematic diagram of the screening process for the OSR-BMSCs. (B to E) The differentially expressed and phosphorylated proteins in OSR-BMSCs were screened using proteomics combined with phosphorylated proteomics under oxidative stress. (B) Differentially phosphorylated site cluster analysis. (C) Differentially expressed protein cluster analysis. (D) Differential phosphorylated site volcano map. (E) Differential protein volcano map. (F to H) Bioinformatics analysis of the combined omics. (F and G) KEGG analysis and GSEA of differentially phosphorylated proteins and enriched signaling pathways related to cell apoptosis and redox. (H) Interaction network analysis of differentially phosphorylated proteins enriched in the redox and apoptosis signaling pathways to screen the crucial regulatory molecule BLK. (I and J) Analysis of the structure of the BLK protein using a predictive tool. (I) 3D structural diagram of the BLK protein molecule. (J) Cysteine residue sites in the kinase domain of BLK.

In previous studies, we confirmed that oxidative stress accompanied the pathogenesis and progression of SONFH (Fig. S1). We found that the redox homeostasis in the osteonecrotic region was disrupted, which resulted in the production of a large amount of ROS (Fig. S1A to K). We transplanted green fluorescent protein (GFP)-labeled BMSCs to repair models of SONFH and noted abundant ROS accumulation in the BMSCs after transplantation (Fig. S1L). About 90% of them underwent apoptosis caused by ROS accumulation (Fig. S1M and N), and the overall efficacy of the transplantation was limited (Fig. S1O to T). However, 10% of the BMSCs were able to survive in the oxidative stress microenvironment induced by osteonecrosis (Fig. S1M). We therefore proceeded to isolate the surviving BMSCs from the transplantation area using the GFP marker. A multicolor flow cytometry analysis then revealed that these surviving BMSCs not only expressed typical BMSC surface antigen markers but also specifically expressed CD72 at a rate of 95% (Fig. 1A and Fig. S2A). We also found a small number of CD72^+^ cells in the primary BMSCs isolated from bone marrow, but the positivity rate was only 11% (Fig. 1A and Fig. S2B). We subsequently labeled primary BMSCs with CD90, isolated CD90^+^/CD72^+^-BMSCs and CD90^+^/CD72^−^-BMSCs via flow cytometry, treated the BMSCs with different concentrations of hydrogen peroxide (H_2_O_2_) to simulate an oxidative stress microenvironment [17,19,21], and then analyzed the resistance of these 2 BMSC subgroups to oxidative stress (Fig. 1A). The results showed that the half-maximal inhibitory concentration (IC_50_) of H_2_O_2_ in the CD90^+^/CD72^+^-BMSCs was 3,000 μM, while that in the CD90^+^/CD72^−^-BMSCs was 600 μM, and their resistance index (RI) was >5 (Fig. S2C and D). The CD90^+^/CD72^+^-BMSCs therefore exhibited resistance to apoptosis induced by oxidative stress, while the CD90^+^/CD72^−^-BMSCs were sensitive to it (Fig. S2E to H). Therefore, to functionally distinguish between the 2 subgroups, we dubbed the CD90^+^/CD72^+^-BMSCs OSR-BMSCs, while the CD90^+^/CD72^−^-BMSCs were dubbed oxidative stress-sensitive BMSCs (OSS-BMSCs).

To explore the regulatory mechanism by which OSR-BMSCs acquire resistance to oxidative stress and thereby resolve the survival issue of transplanted BMSCs in the oxidative stress microenvironment, we screened the redox and apoptotic signaling networks in OSR-BMSCs by combining quantitative and phosphorylated proteomic analyses (Fig. 1A). The results showed that a total of 528 differentially expressed phosphorylated sites were identified by phosphorylated proteomics (corresponding to 207 proteins; fold change > 2, false discovery rate (FDR) < 1%, *P* < 0.05; Fig. 1B and D), and 106 differentially expressed proteins were identified by proteomics (fold change > 2, FDR < 1%, *P* < 0.05; Fig. 1C and E). A Kyoto Encyclopedia of Genes and Genomes (KEGG) assessment and gene set enrichment analysis (GSEA) revealed that the differentially phosphorylated proteins were significantly enriched in redox and apoptotic signaling pathways such as AMP-activated protein kinase (AMPK), Akt, FOXO, NF-κB, ERK, and signal transducer and activator of transcription (STAT) (Fig. 1F and G). To screen out the crucial factors related to the cross-linking between the redox and apoptotic signaling networks, we performed a kinase substrate interaction network analysis of the identified pathways. The results showed that BLK was at the core of the network and shared a common expression pattern with the downstream effector proteins of each signaling pathway (Fig. 1H). The PredictProtein platform predicted that the amino acids at 313, 367, 368, 450, 454, and 466 in the kinase domain of BLK were cysteine residues (Fig. 1I and J). Previous studies have shown that cysteine residues can be oxidized by ROS to undergo covalent modification and that covalent modification of BLK is a necessary condition for activating auto-tyrosine kinase activity [40,41]. Therefore, BLK has the structural basis to directly respond to intracellular ROS changes. Based on our bioinformatic analysis, we verified the combined omics data in BMSCs. The results showed that BLK expression and phosphorylation were up-regulated in OSR-BMSCs in an environment without oxidative stress (Fig. S3A to D). Likewise, the phosphorylation levels of STAT3, Akt, FOXO3a, NF-κB, AMPK, and ERK1/2 (Fig. S3E to Q), as well as downstream effector proteins such as CAT, SOD, Bcl-2, and Survivin, were increased (Fig. S3R to V). The stimulation of oxidative stress further up-regulated the expression of BLK, p-BLK, p-STAT3, p-Akt, p-FOXO3a, p-NF-κB, p-AMPK, p-ERK1/2, CAT, SOD, Bcl-2, and Survivin (Fig. S3). These results suggest that BLK may be involved in the process whereby OSR-BMSCs acquire resistance to oxidative stress.

### Knockdown of BLK eliminates the resistance of OSR-BMSCs to oxidative stress and leads to stress-induced apoptosis

**Fig. 2.**
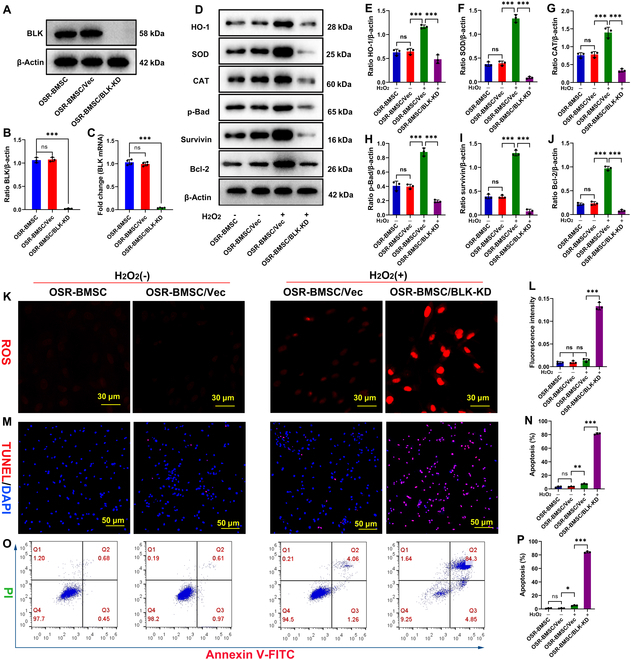
BLK knockdown eliminates the oxidative stress resistance of OSR-BMSCs and aggravates their stress-induced apoptosis. Knockdown of BLK in OSR-BMSCs using shRNA interference technology. (A and B) The expression of BLK protein was detected by Western blot (*n* = 4). (C) The expression of BLK mRNA was detected by qPCR (*n* = 4). After the knockdown of BLK, OSR-BMSCs were treated with 1,000 μM H_2_O_2_ to simulate oxidative stress microenvironment for 24 h, and the antioxidant and antiapoptotic abilities of OSR-BMSCs were analyzed. (D to J) The expression levels of antioxidants HO-1, MnSOD, and CAT, and apoptosis-related proteins p-Bad, Survivin, and Bcl-2 were detected by Western blot (*n* = 4). (K and L) The ROS levels were detected by DHE fluorescent probe (*n* = 4). (M and N) The cells with DNA damage were detected by TUNEL staining (*n* = 4). (O and P) Detection of apoptosis by flow cytometry (*n* = 4). All data are presented as the means ± SD. (B, C, E to J, L, N, and P) One-way ANOVA with Tukey’s post hoc tests was used for statistical analysis, *P < 0.05, **P < 0.01, ***P < 0.001. Vec, empty vector; BLK-KD, BLK knockdown; H_2_O_2_(+), hydrogen peroxide treatment; H_2_O_2_(−), no treatment with hydrogen peroxide.

We found that BLK expression was up-regulated in OSR-BMSCs and was further up-regulated by oxidative stress stimulation (Fig. S3A). Therefore, we designed a short hairpin RNA (shRNA) for BLK mRNA and used RNA interference technology to knock down BLK expression in OSR-BMSCs. Successful knockdown of BLK was confirmed via quantitative polymerase chain reaction (qPCR) and Western blot (Fig. 2A to C). We then exposed OSR-BMSCs to a simulated oxidative stress microenvironment of 1,000 μM H_2_O_2_ for 24 h. The expression levels of the antioxidant enzymes HO-1, SOD, and CAT, as well as the antiapoptotic proteins Survivin and Bcl-2, were found to be significantly up-regulated after oxidative stress (Fig. 2D to J). There were no significant changes in ROS content, DNA damage, and apoptosis rate (Fig. 2K to P)—indicating that the OSR-BMSCs were able to resist oxidative stress damage. When BLK was knocked down in OSR-BMSCs, the expression levels of HO-1, SOD, CAT, p-Bad, Survivin, and Bcl-2 were significantly down-regulated (Fig. 2D to J), ROS content increased (Fig. 2K and L), the percentage of TUNEL^+^ (terminal deoxynucleotidyl transferase-mediated deoxyuridine triphosphate nick end labeling-positive) cells increased, and the apoptosis rate accelerated significantly (Fig. 2M to P). These results show that knockdown of BLK eliminates the ability of OSR-BMSCs to resist oxidative stress.

Similarly, we detected the expression of BLK in oxidative stress-resistant human BMSCs (OSR-hBMSCs) and further verified the role of BLK in them. We knocked down the expression of BLK in OSR-hBMSCs (Fig. S4A to C) and then exposed the cells to 1,000 μM H_2_O_2_ for 24 h. The results confirmed that the expression of antioxidant enzymes and anti-apoptotic proteins in OSR-hBMSCs increased under oxidative stress (Fig. S4D to J), and there were no significant changes in terms of intracellular ROS, DNA damage, and cell apoptosis (Fig. S4K to P). After BLK knockdown in OSR-hBMSCs, the expression levels of HO-1, SOD, CAT, p-Bad, Survivin, and Bcl-2 were significantly down-regulated (Fig. S4D to J), and the ROS level and apoptosis rate increased even further (Fig. S4K to P). These results confirm that BLK knockdown also caused OSR-hBMSCs to lose resistance to oxidative stress.

### Overexpression of BLK enables OSS-BMSCs to acquire resistance to oxidative stress and thus resist stress-induced apoptosis

**Fig. 3.**
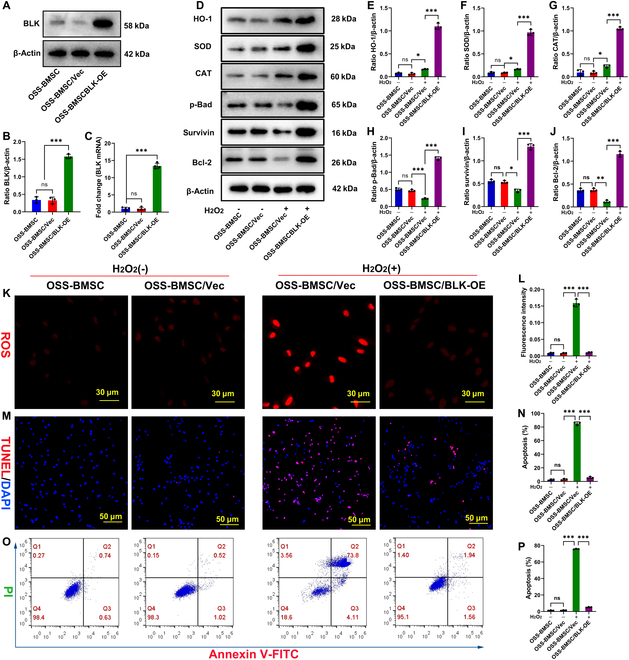
Overexpression of BLK enables OSS-BMSCs to acquire resistance to oxidative stress and thus resist stress-induced apoptosis. Overexpression of BLK in OSS-BMSCs using gene transfection overexpression technology. *(*A and B) The expression of BLK protein was detected by Western blot (*n* = 4). (C) The expression of BLK mRNA was detected by qPCR (*n* = 4). After up-regulating BLK, OSS-BMSCs were treated with 1,000 μM H_2_O_2_ for 24 h to analyze the antioxidant and antiapoptotic properties of the OSS-BMSCs. (D to J) The expression levels of the antioxidants HO-1, MnSOD, and CAT and the apoptosis-related proteins p-Bad, Survivin, and Bcl-2 were detected by Western blot (*n* = 4). (K and L) ROS levels were detected by DHE fluorescence probe (*n* = 4). (M and N) The cells with DNA damage were detected by TUNEL staining (*n* = 4). (O and P) Detection of apoptosis by flow cytometry (*n* = 4). All data are presented as the means ± SD. (B, C, E to J, L, N, and P) One-way ANOVA with Tukey’s post hoc tests was used for statistical analysis, *P < 0.05, **P < 0.01, ***P < 0.001. BLK-OE, BLK overexpression.

BLK is expressed at low levels in OSS-BMSCs (Fig. S3A). Therefore, a question arises regarding whether overexpression of BLK in OSS-BMSCs would enable the cells to acquire resistance to oxidative stress and thus resist stress-induced apoptosis. To assess this, we overexpressed BLK in OSS-BMSCs using gene transfection. The overexpression of BLK was confirmed via qPCR and Western blot, and the cells were then exposed to 1,000 μM H_2_O_2_ to simulate an oxidative stress microenvironment, for 24 h (Fig. 3A to C). The expression levels of the antioxidant enzymes HO-1, CAT, and SOD were found to be slightly up-regulated in response to oxidative stress, whereas those of Bcl-2, Survivin, and p-Bad were significantly down-regulated (Fig. 3D to J). The ROS content and proportion of TUNEL^+^ cells were also increased (Fig. 3K to N), and stress-induced apoptosis exceeded 70% (Fig. 3O and P). Following BLK overexpression in OSS-BMSCs, we found that the levels of HO-1, CAT, and SOD were significantly up-regulated; the levels of Survivin, Bcl-2, and p-Bad were also up-regulated (Fig. 3D to J); the intracellular ROS content and proportion of TUNEL^+^ cells decreased (Fig. 3K to N); and stress-induced apoptosis was significantly reduced (Fig. 3O and P). These results show that the overexpression of BLK enables OSS-BMSCs to acquire resistance to oxidative stress and thus resist stress-induced apoptosis.

Similar results were obtained in human BMSCs. We detected the expression of BLK in oxidative stress-sensitive human BMSCs (OSS-hBMSCs) and further validated the role of BLK in these cells. We overexpressed BLK in OSS-hBMSCs (Fig. S5A to C) and then treated cells with 1,000 μM H_2_O_2_ for 24 h. Under oxidative stress conditions, overexpression of BLK up-regulated the expression of antioxidant enzymes and antiapoptotic proteins (Fig. S5D to J) and decreased ROS levels as well as stress-induced apoptosis (Fig. S5K to P). These results confirm that overexpression of BLK can allow OSS-hBMSCs to resist stress-induced apoptosis.

### Overexpression of BLK improves the survival and therapeutic efficacy of transplanted OSS-BMSCs

**Fig. 4.**
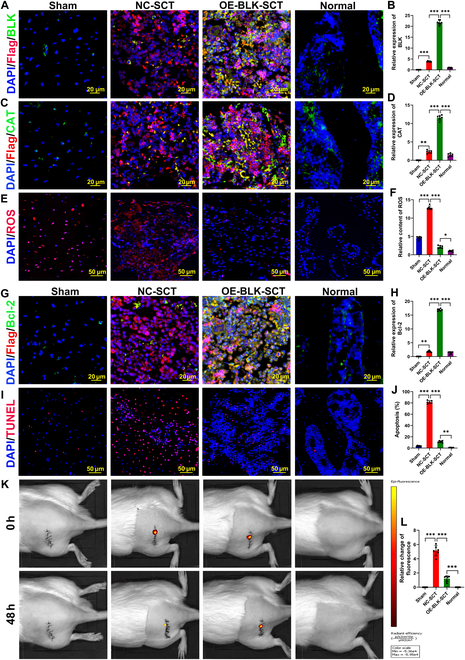
BLK overexpression promotes the survival of transplanted OSS-BMSCs. Tissue-engineered bone was constructed with OSS-BMSCs that overexpressed BLK and then transplanted to repair early-stage SONFH in an animal model. At 48 h after transplantation: (A and B) Immunofluorescence staining of BLK expression levels in OSS-BMSCs (*n* = 6; flag not only reflects BLK expression levels but also labels the transplanted OSS-BMSCs). (C and D) CAT immunofluorescence staining in OSS-BMSCs (*n* = 6). (E and F) ROS levels in the transplantation area were detected using a DHE fluorescence probe (*n* = 6). (G and H) Bcl-2 immunofluorescence staining in OSS-BMSCs (*n* = 6). (I and J) Apoptosis of transplanted OSS-BMSCs was detected by TUNEL (*n* = 6). (K and L) Survival of transplanted OSS-BMSCs was detected via small animal imaging (*n* = 6). All data are presented as the means ± SD. (A, D, F, H, J, and L) One-way ANOVA with Tukey’s post hoc tests was used for statistical analysis, *P < 0.05, **P < 0.01, ***P < 0.001. NC-SCT, negative controlled-stem cell transplantation; OE-BLK-SCT, BLK-overexpressed stem cell transplantation.

Our previous results have confirmed that the osteonecrotic region of SONFH is an oxidative stress microenvironment (Fig. S1). To verify the pro-survival effect of BLK in this setting in vivo, we labeled OSS-BMSCs with the cell membrane near-infrared fluorescent probe DiR and used them to construct tissue-engineered bone to repair SONFH in rats. Consistent with our in vitro results, overexpression of BLK increased the expression levels of BLK and CAT in the OSS-BMSCs 48 h after their transplantation (Fig. 4A to D). It also decreased the ROS level in the transplanted area (Fig. 4E and F), increased the expression of Bcl-2 in the OSS-BMSCs (Fig. 4G and H), significantly decreased the proportion of TUNEL^+^ cells (Fig. 4I and J), and significantly increased the surviving OSS-BMSCs in the transplanted region (Fig. 4K and L). These results confirm that BLK can mediate the acquisition of oxidative stress resistance by OSS-BMSCs and the resultant inhibition of stress-induced apoptosis, thereby improving the survival of transplanted OSS-BMSCs.

**Fig. 5.**
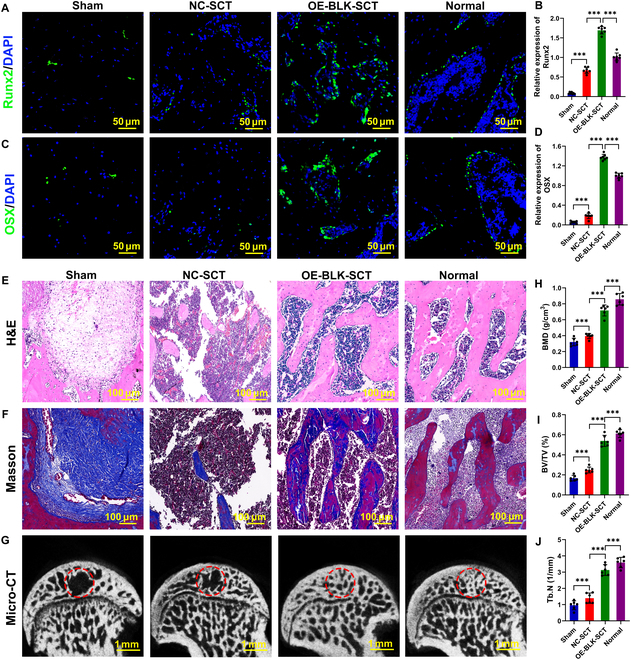
Overexpression of BLK in OSS-BMSCs improves the repair efficacy of tissue-engineered bone for early SONFH. Tissue-engineered bone was constructed using OSS-BMSCs that overexpressed BLK and then transplanted to repair early-stage SONFH in an animal (rat) model. The repair efficacy of osteonecrosis areas was evaluated at 12 weeks after the transplantation. (A to D) The expression levels of Runx2 and OSX in bone grafts were detected by immunofluorescence (*n* = 6). (E) The repair efficiency of the graft area was detected by H&E (*n* = 6). (F) The maturity of the new bone at the bone graft area was evaluated by Masson (*n* = 6). (G to J) The repair efficiency of the bone defect was evaluated by micro-CT (*n* = 6). All data are presented as the means ± SD. (B, D, and H to J) One-way ANOVA with Tukey’s post hoc tests was used for statistical analysis, *P < 0.05, **P < 0.01, ***P < 0.001.

In light of the above, we also evaluated the effect of tissue-engineered bone constructed by BLK-modified OSS-BMSCs on early SONFH repair. An immunofluorescence analysis at 12 weeks after OSS-BMSC transplantation showed a slight increase in the number of Runx2 and OSX double-positive cells in the transplantation area of the negative control stem cell transplantation group (Fig. 5A to D). Hematoxylin and eosin (H&E) and Masson staining showed that new bone tissue had been formed by crawling replacement around the xenogeneic antigen-extracted cancellous bone (XACB) in the transplantation area, and reactive bone hyperplasia was observed around the transplantation area (Fig. 5E and F). Micro-computed tomography (CT) analysis showed a small quantity of new bone trabeculae in the transplantation area, with slightly increased bone mineral density (BMD), bone volume fraction (BVF), trabecular number (Tb.N), and trabecular thickness (Tb.Th; Fig. 5H to J), but a persistent presence of large bone defects (Fig. 5G). However, overexpression of BLK resulted in a higher number of Runx2 and OSX double-positive cells in the transplantation area (Fig. 5A to D). H&E and Masson staining showed no obvious bone defects, and there were a large number of immature new bone tissue zones in the transplantation area (Fig. 5E and F). Micro-CT analysis showed new bone trabeculae in the transplantation area, which were thinner and irregularly arranged (Fig. 5G), with significantly increased BMD, BVF, Tb.N, and Tb.Th (Fig. 5H to J). These results indicate that overexpression of BLK to promote BMSC survival can improve the therapeutic effect of tissue-engineered bone on early SONFH.

### BLK knockdown decreases the survival and repair efficacy of transplanted OSR-BMSCs

**Fig. 6.**
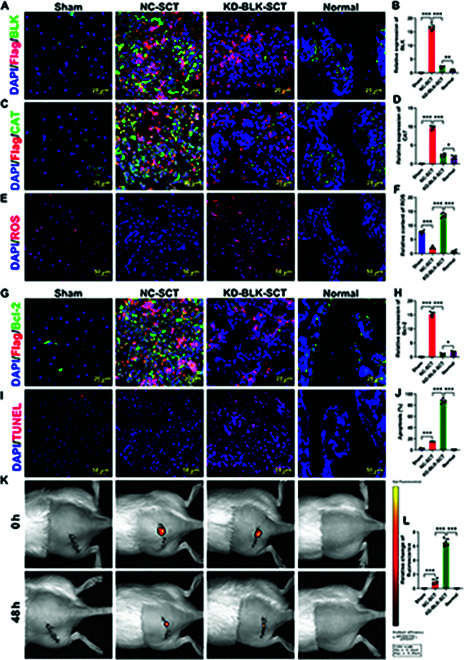
BLK knockdown decreases the survival of transplanted OSR-BMSCs. Tissue-engineered bone was constructed with OSR-BMSCs that knocked down BLK and then transplanted to repair early-stage SONFH. At 48 h after transplantation: (A and B) Immunofluorescence staining of BLK expression levels in OSR-BMSCs (*n* = 6). (C and D) CAT immunofluorescence staining in OSR-BMSCs (*n* = 6). (E and F) ROS levels in the transplanted area were detected by the DHE fluorescence probe (*n* = 6). (G and H) Bcl-2 immunofluorescence staining in OSR-BMSCs (*n* = 6). (I and J) Apoptosis of transplanted OSS-BMSCs was detected by TUNEL (*n* = 6). (K and L) Survival of transplanted OSS-BMSCs was detected by small animal imaging (*n* = 6). All data are presented as the means ± SD. (A, D, F, H, J, and L) One-way ANOVA with Tukey’s post hoc tests was used for statistical analysis, *P < 0.05, **P < 0.01, ***P < 0.001. KD-BLK-SCT, BLK-knockdown stem cell transplantation.

Next, we knocked down BLK in OSR-BMSCs and once again verified the effect of its down-regulation on the survival of transplanted OSR-BMSCs. We constructed tissue-engineered bone using DiR-labeled OSR-BMSCs and transplanted it to repair early-stage SONFH in an animal model. At 48 h after OSR-BMSC transplantation, we found that the expression levels of CAT and Bcl-2 were significantly down-regulated in the BLK-knockdown OSR-BMSCs (Fig. 6A to D, G, and H), while the ROS levels (Fig. 6E and F) and proportion of TUNEL^+^ cells (Fig. 6I and J) were increased. In vivo imaging of the animals showed a significant reduction in the number of surviving OSR-BMSCs in the transplantation area (Fig. 6K to L). These results confirmed that BLK knockdown eliminates the ability of OSR-BMSCs to resist oxidative stress and makes it difficult for the cells to survive in the oxidative stress microenvironment of the osteonecrosis area.

**Fig. 7.**
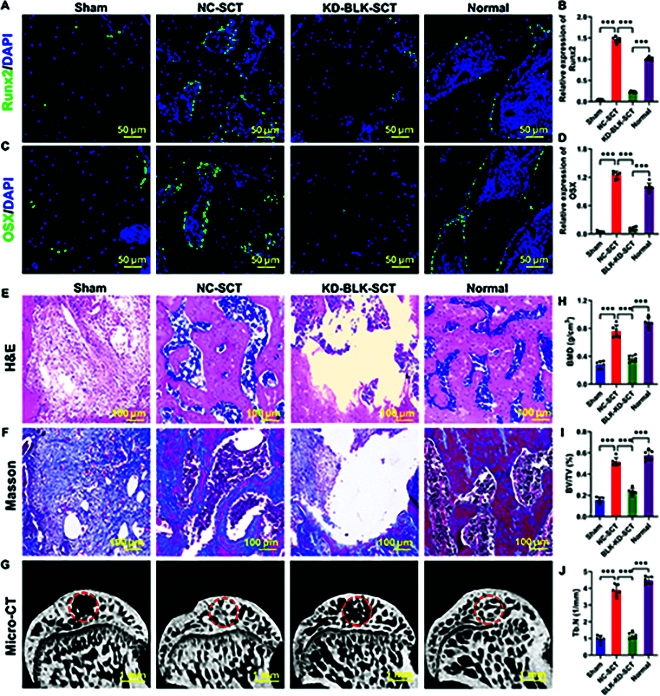
BLK knockdown in OSR-BMSCs limits the repair efficacy of tissue-engineered bone for early SONFH. Tissue-engineered bone was constructed using OSR-BMSCs that knocked down BLK and then transplanted to repair early-stage SONFH. The repair efficacy of osteonecrosis areas was evaluated at 12 weeks after the transplantation. (A to D) The expression levels of Runx2 and OSX in bone grafts were detected by immunofluorescence (*n* = 6). (E) The repair efficiency of the graft area was detected by H&E (*n* = 6). (F) The maturity of the new bone at the bone graft area was evaluated by Masson (*n* = 6). (G to J) The repair efficiency of the bone defect was evaluated by micro-CT (*n* = 6). All data are presented as the means ± SD. (B, D, and H to J) One-way ANOVA with Tukey’s post hoc tests was used for statistical analysis, *P < 0.05, **P < 0.01, ***P < 0.001.

We continued evaluating the effect of tissue-engineered bone constructed by BLK-knockdown OSR-BMSCs on the repair of early-stage SONFH in our animal model. At 12 weeks after OSR-BMSC transplantation, immunofluorescence showed a large number of Runx2 and OSX double-positive cells in the transplantation area of the negative control group (Fig. 7A to D). H&E and Masson staining showed a large amount of new bone tissue zones in the transplantation area, and the bone defects had been almost repaired (Fig. 7E and F). Micro-CT analysis showed new bone trabeculae in the transplantation area, with irregular arrangement of bone trabeculae (Fig. 7G), as well as significantly increased levels of BMD, BVF, Tb.N, and Tb.Th (Fig. 7H to J). However, BLK knockdown resulted in a significant decrease in the number of Runx2 and OSX double-positive cells in the transplantation area (Fig. 7A to D). H&E and Masson staining showed obvious bone defects, and the new bone tissue zones were only observed around the bone defect area (Fig. 7E and F). Micro-CT revealed a small amount of new bone trabeculae at the transplantation site, as well as significantly decreased levels of BMD, BVF, Tb.N, and Tb.Th (Fig. 7G to J). These results suggest that BLK knockdown reduces the survival of transplanted OSR-BMSCs and limits the efficacy of tissue-engineered bone for treating early-stage SONFH.

### BLK targets activation of the ERK1/2, Akt, STAT3, and NF-κB signaling pathways

To further reveal the mechanism by which BLK regulates the acquisition of oxidative stress resistance by OSR-BMSCs, we performed a phosphorylated proteomic analysis of OSR-BMSCs with BLK knockdown and OSS-BMSCs with BLK overexpression to screen the signaling pathways regulated by BLK (Fig. S6A and D). After BLK knockdown in OSR-BMSCs, the differentially expressed phosphorylated proteins were found to be primarily enriched in the apoptosis, ERK, Akt, STAT, NF-κB, and p53 signaling pathways (Fig. S6B and C). After overexpression of BLK in OSS-BMSCs, the differentially expressed phosphorylated proteins were primarily enriched in the ERK, Akt, STAT, NF-κB, AMPK, and FOXO signaling pathways (Fig. S6E and F). Thus, ERK, Akt, STAT, and NF-κB may be the downstream signaling pathways regulated by BLK.

**Fig. 8.**
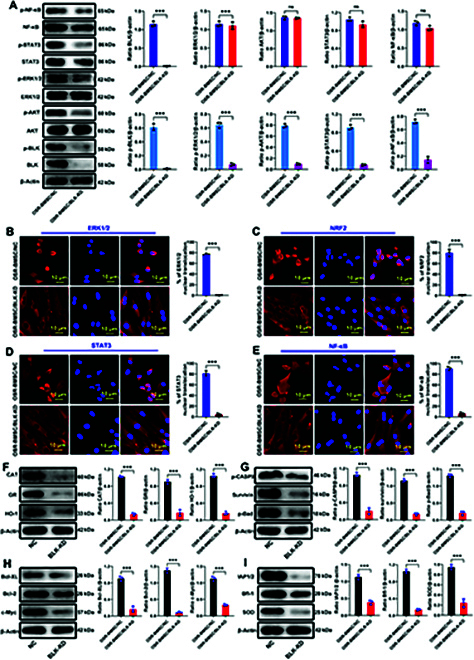
Knockdown of BLK inhibits the activation of the ERK1/2, Akt, STAT3, and NF-κB signaling pathways. Statuses of the ERK1/2, Akt, STAT3, and NF-κB signaling pathways were detected after knocking down BLK in OSR-BMSCs. (A) The protein and phosphorylation levels of ERK1/2, Akt, STAT3, and NF-κB were detected by Western blot (*n* = 3). (B to E) The nuclear translocation of ERK1/2, NRF2, STAT3, and NF-κB was detected by immunofluorescence (*n* = 3). (F) The expression levels of the ERK signaling pathway effector proteins (CAT, GR, and HO-1) were detected by Western blot (*n* = 3). (G) The expression levels of the Akt signaling pathway effector proteins (p-CASP9, Survivin, and p-Bad) were detected by Western blot (*n* = 3). (H) The expression levels of the STAT signaling pathway effector proteins (Bcl-XL, Bcl-2, and c-Myc) were detected by Western blot (*n* = 3). (I) The expression levels of the NF-κB signaling pathway effector proteins (IAP1/2, Bfl-1, and SOD) were detected by Western blot (*n* = 3). All data are presented as the means ± SD. (A to I) The statistical analysis was performed by unpaired *t* test, *P < 0.05, **P < 0.01, ***P < 0.001.

**Fig. 9.**
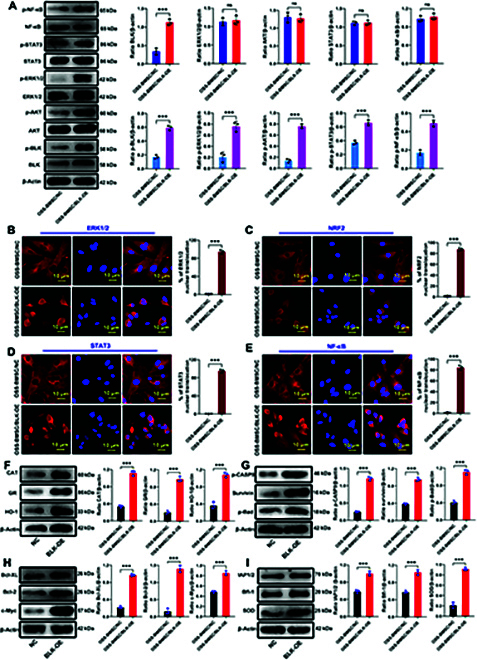
Overexpression of BLK activates the ERK1/2, Akt, STAT3, and NF-κB signaling pathways. Statuses of the ERK1/2, Akt, STAT3, and NF-κB signaling pathways were detected after overexpressing BLK in OSS-BMSCs. (A) The protein and phosphorylation levels of ERK1/2, Akt, STAT3, and NF-κB were detected by Western blot (*n* = 3). (B to E) The nuclear translocation of ERK1/2, NRF2, STAT3, and NF-κB was detected by immunofluorescence (*n* = 3). (F) The expression levels of the ERK signaling pathway effector proteins (CAT, GR, and HO-1) were detected by Western blot (*n* = 3). (G) The expression levels of the Akt signaling pathway effector proteins (p-CASP9, Survivin, and p-Bad) were detected by Western blot (*n* = 3). (H) The expression levels of the STAT signaling pathway effector proteins (Bcl-XL, Bcl-2, and c-Myc) were detected by Western blot (*n* = 3). (I) The expression levels of the NF-κB signaling pathway effector proteins (IAP1/2, Bfl-1, and SOD) were detected by Western blot (*n* = 3). All data are presented as the means ± SD. (A to I) The statistical analysis was performed by unpaired *t* test, *P < 0.05, **P < 0.01, ***P < 0.001.

Next, we verified the regulatory effect of BLK on the ERK, Akt, STAT, and NF-κB signaling pathways. BLK knockdown in OSR-BMSCs significantly reduced the phosphorylation levels of ERK1/2, Akt, STAT3, and NF-κB (Fig. 8A), decreased the nuclear translocation of ERK1/2, NRF2, STAT3, and NF-κB (Fig. 8B to E), and significantly down-regulated the expression levels of antioxidant and antiapoptotic proteins downstream of these pathways (Fig. 8F to I). By contrast, BLK overexpression in OSS-BMSCs significantly increased the phosphorylation levels of ERK1/2, Akt, STAT3, and NF-κB (Fig. 9A), increased the nuclear translocation of ERK1/2, NRF2, STAT3, and NF-κB (Fig. 9B to E), and up-regulated the expression levels of antioxidant and antiapoptotic proteins downstream of these pathways (Fig. 9F to I). These results confirmed that BLK promotes the expression of antioxidant and antiapoptotic proteins by cross-linking and activating the ERK1/2, Akt, STAT3, and NF-κB signaling pathways.

### BLK inhibits stress-induced apoptosis of BMSCs by targeting activation of the ERK1/2, Akt, STAT3, and NF-κB signaling pathways

**Fig. 10.**
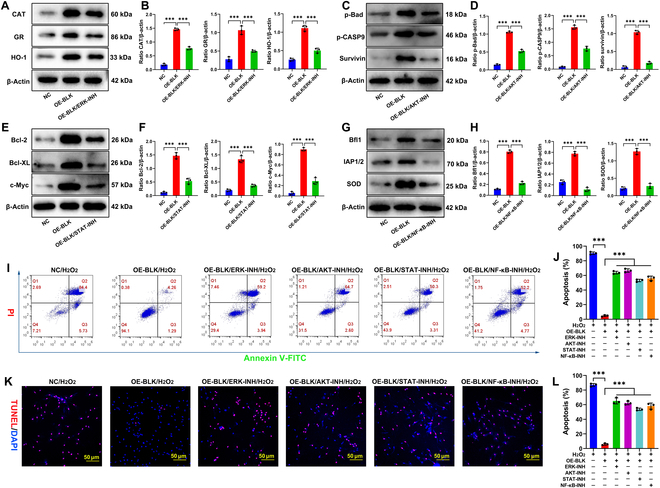
Blocking of the ERK1/2, Akt, STAT3, and NF-κB signaling pathways weakens the role of BLK in resistance to stress-induced apoptosis. After overexpressing BLK in OSS-BMSCs, specific inhibitors were applied to block the ERK1/2, Akt, STAT3, and NF-κB signaling pathways, separately. (A and B) The expression levels of the ERK1/2 signaling pathway effector proteins (CAT, GR, and HO-1) were detected by Western blot (*n* = 3). (C and D) The expression levels of the Akt signaling pathway effector proteins (p-CASP9, Survivin, and p-Bad) were detected by Western blot (*n* = 3). (E and F) The expression levels of the STAT3 signaling pathway effector proteins (Bcl-XL, Bcl-2, and c-Myc) were detected by Western blot (*n* = 3). (G and H) The expression levels of the NF-κB signaling pathway effector proteins (IAP1/2, Bfl-1, and SOD) were detected by Western blot (*n* = 3). Effects of blocking the ERK1/2, Akt, STAT3, and NF-κB signaling pathways on BLK resistance to stress-induced apoptosis. (I and J) Apoptosis was detected by flow cytometry (*n* = 4). (K and L) Detection of the proportion of TUNEL-positive cells (*n* = 4). All data are presented as the means ± SD. (B, D, F, H, J, and L) One-way ANOVA with Tukey’s post hoc tests was used for statistical analysis, *P < 0.05, **P < 0.01, ***P < 0.001. INH, inhibitor.

**Fig. 11.**
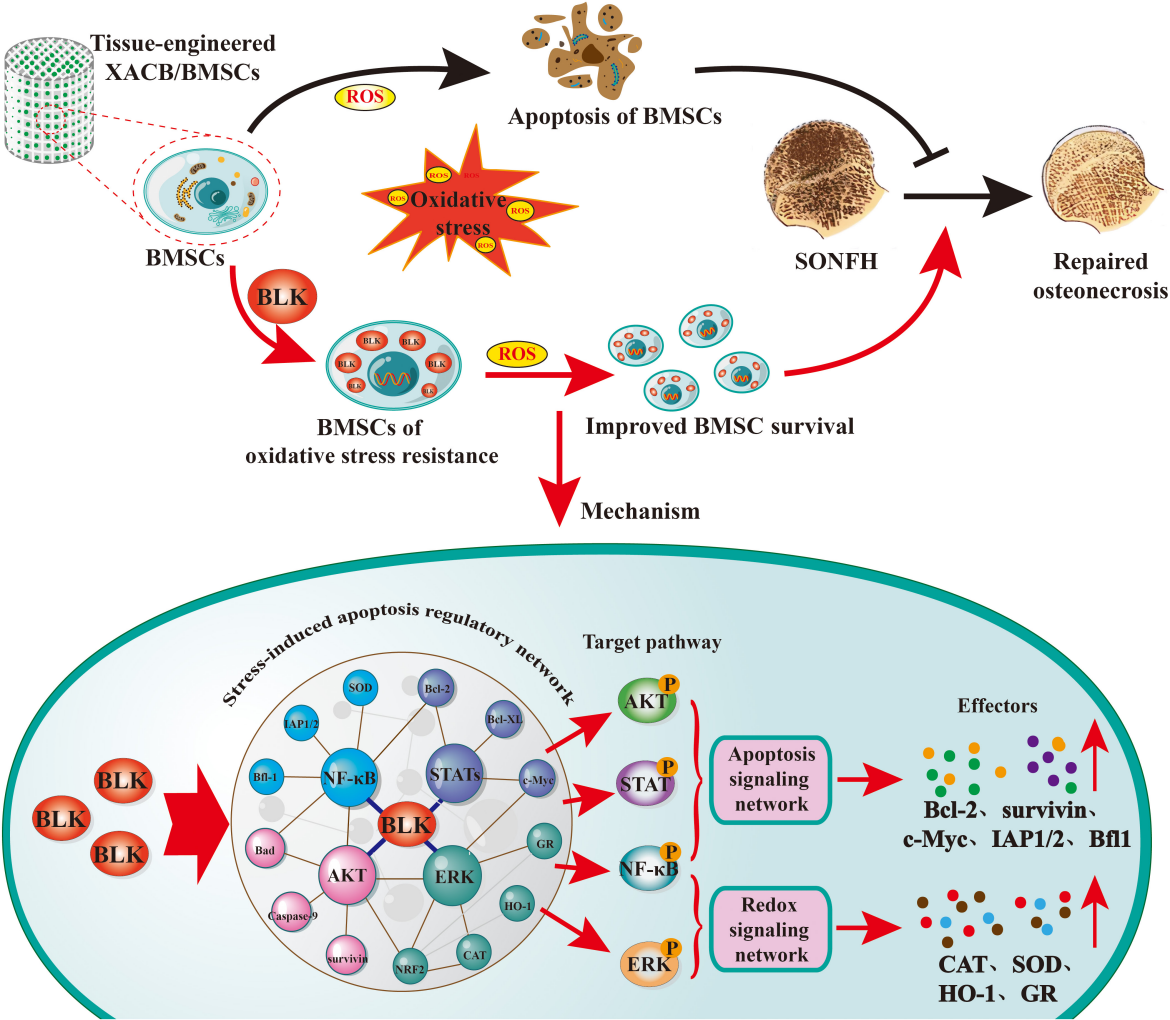
BLK cross-links and activates the redox and apoptotic signaling networks, mediating BMSCs to acquire resistance to oxidative stress and avoid stress-induced apoptosis—which improves the repair efficiency of tissue-engineered bone for early SONFH. The osteonecrotic area of SONFH is an oxidative stress microenvironment. When tissue-engineered bone constructed with BMSCs is used to repair SONFH, the transplanted BMSCs undergo stress-induced apoptosis in the osteonecrotic area, which limits the efficiency of the tissue-engineered bone in terms of repairing SONFH. B-lymphotyrosine kinase (BLK) cross-links and activates redox and apoptotic signaling networks such as ERK1/2, Akt, STAT3, and NF-κB to up-regulate the expression of antioxidant enzymes and antiapoptotic proteins, which can lead BMSCs to acquire resistance to oxidative stress, promote the survival of transplanted BMSCs, and improve the repair effect of tissue-engineered bone on SONFH.

To further investigate the roles of the ERK, Akt, STAT, and NF-κB signaling pathways in the BLK regulation of stress-induced BMSC apoptosis, we overexpressed BLK in OSS-BMSCs. This significantly increased the levels of intracellular antioxidant enzymes and antiapoptotic proteins such as SOD, CAT, GR, HO-1, Bcl-2, Bcl-XL, IAP/1/2, Survivin, c-Myc, Bfl1, p-Bad, and p-CASP9 (Fig. 10A to H), allowing the cells to resist stress-induced apoptosis (Fig. 10I to L). Next, based on BLK overexpression in OSS-BMSCs, we used signal inhibitors to block the ERK1/2, Akt, STAT3, and NF-κB pathways, separately. Western blot analysis showed that blocking the ERK1/2 signaling pathway down-regulated the expression of antioxidants such as CAT, GR, and HO-1 (Fig. 10A and B); blocking the Akt signaling pathway down-regulated the expression of Survivin, p-Bad, and p-CASP9 (Fig. 10C and D); blocking the STAT3 signaling pathway down-regulated the expression of Bcl-2, Bcl-XL, and c-Myc (Fig. 10E to F); and blocking the NF-κB signaling pathway down-regulated the expression of Bfl1, IAP/1/2, and MnSOD (Fig. 10G and H). Moreover, blocking the ERK1/2, Akt, STAT3, and NF-κB signaling pathways resulted in a significant increase in stress-induced apoptosis (*P* < 0.05; Fig. 10I to L). These results confirm that BLK inhibits stress-induced apoptosis in BMSCs by targeting the ERK1/2, Akt, STAT3, and NF-κB signaling pathways (Fig. 11).

## Discussion

In this study, we identified a new subgroup of BMSCs with resistance to oxidative stress (i.e., OSR-BMSCs). Based on our findings, BLK is the crucial molecule that regulates the abilities of both human and rat BMSCs to acquire resistance to oxidative stress. The differential expression of BLK mediates the different tolerance of BMSCs to oxidative stress. BLK may act as an oxidative stress sensor to respond to the changes in intracellular ROS and then cross-link and activate the ERK, Akt, STAT, and NF-κB signaling pathways by changing its own tyrosine kinase activity to synergically regulate the redox and apoptotic signaling networks—thereby enabling BMSCs to acquire resistance to oxidative stress and resist stress-induced apoptosis. To the best of our knowledge, this is the first report describing the role of OSR-BMSCs and BLK in stress-induced apoptosis, thus providing a new research strategy and regulatory target for improving the survival and therapeutic efficacy of transplanted BMSCs.

Oxidative stress is involved in the pathogenesis and progression of many diseases, including SONFH, and is also a major factor restricting the utility of BMSC transplantation for their treatment [15,42–45]. In our animal model of SONFH, we confirmed that a variety of oxidative stress markers—such as ROS, NOX4, 8-OH-dG, and MDA—were increased in the pathogenesis and progression of SONFH. We found that 48 h after BMSCs were transplanted to repair the animal-model SONFH, the oxidative stress markers and DNA damage in the cells were increased, and a large number of transplanted BMSCs underwent apoptosis—thus severely limiting the transplantation efficacy. This result is consistent with previous literature [27–30,46]. It should be emphasized that the oxidative stress microenvironment at the lesion site hinders the clinical application of BMSCs. When analyzing how to overcome this obstacle, we found notable differences in the adaptability of our transplanted BMSCs to the oxidative stress microenvironment. Although a large number of them underwent stress-induced apoptosis after transplantation, some were consistently able to survive in the oxidative stress microenvironment of the osteonecrotic area. Our research confirmed that these surviving BMSCs expressed CD72 specifically. Noting this characteristic, we labeled primary BMSCs with CD90 and then isolated CD90^+^/CD72^+^-BMSCs and CD90^+^/CD72^−^-BMSCs by flow cytometry. The CD90^+^/CD72^−^-BMSCs were found to be sensitive to oxidative stress and exhibited ROS accumulation and stress-induced apoptosis in the H_2_O_2_-simulated oxidative stress microenvironment. By contrast, the CD90^+^/CD72^+^-BMSCs were resistant to oxidative stress and showed significantly reduced ROS accumulation and stress-induced apoptosis under oxidative stress. Therefore, we dubbed the CD90^+^/CD72^−^-BMSCs “OSS-BMSCs” and the CD90^+^/CD72^+^-BMSCs “OSR-BMSCs”. To resolve the issue of stress-induced apoptosis of transplanted BMSCs, previous studies mainly focused on the stress-induced apoptosis process and apoptotic BMSCs. By contrast, this study focused on the BMSCs that survived in the oxidative stress microenvironment and thus discovered a new subgroup of BMSCs (the OSR-BMSCs) from which we identified the crucial factor (BLK) that regulates the ability of BMSCs to acquire resistance to oxidative stress.

According to recent relevant research, the biological functions of BLK mainly involve cell differentiation, proliferation, migration, and immune regulation, whereas the regulatory role of BLK in stress-induced apoptosis has not been reported [47–50]. In this study, we found that the expression levels of BLK in rat OSR-BMSCs were significantly higher than those in OSS-BMSCs, under both normal and oxidative stress conditions. BLK knockdown in OSR-BMSCs decreased resistance to oxidative stress and increased stress-induced apoptosis, whereas BLK overexpression improved the ability of OSS-BMSCs to resist oxidative stress and effectively inhibited stress-induced apoptosis. Similar effects were found in hBMSCs. Moreover, the therapeutic effect of BMSC transplantation in our animal model showed that BLK overexpression enabled the OSS-BMSCs to acquire resistance to oxidative stress, which effectively improved their survival following transplantation into the osteonecrotic area, and promoted the repair effect of OSS-BMSCs on SONFH. However, after knocking down BLK in OSR-BMSCs, a large number of the transplanted OSR-BMSCs showed stress-induced apoptosis, which seriously limited their effect on SONFH. Therefore, this study highlights a new role of BLK in regulating stress-induced apoptosis—potentially providing a new regulatory target for improving the survival and efficacy of transplanted BMSCs under conditions involving oxidative stress.

In terms of the regulatory mechanism, stress-induced apoptosis involves 2 crucial biological processes: redox and apoptosis [51–53]. Therefore, current interventions related to stress-induced apoptosis mainly focus on maintaining the intracellular redox balance or blocking the activation of apoptotic signal cascades, thereby playing antioxidant or antiapoptotic roles, respectively. However, it is difficult for a single regulatory mechanism to both maintain the redox balance and block the activation of apoptosis [54–58]. Here, we confirmed a novel role of BLK in terms of regulating stress-induced apoptosis. Previous studies have shown that BLK has tyrosine kinase activity and can regulate various signaling pathways through protein phosphorylation. To reveal the mechanism by which BLK regulates BMSCs to acquire resistance to oxidative stress and thus resist stress-induced apoptosis, we conducted a phosphorylated proteomic analysis. The results showed that when BLK was overexpressed or knocked down in BMSCs, the differentially phosphorylated proteins were mainly enriched in the ERK, Akt, STAT, and NF-κB signaling pathways. These pathways are known to be the main mediators of anti-oxidation and anti-apoptosis. Our blocking experiment further confirmed that the inhibitory effect of BLK on stress-induced apoptosis was weakened when the ERK, Akt, STAT, or NF-κB signaling pathways were blocked. These data suggest that BLK is different from ordinary antioxidant and antiapoptotic molecules, because it can mediate the acquisition of oxidative stress resistance in BMSCs through the synergistic activation of the ERK, Akt, STAT, and NF-κB signaling networks—thereby allowing them to resist stress-induced apoptosis.

Starting from OSR-BMSCs represented the major characteristic and strength of this study. However, there were also certain shortcomings worth noting. For example, our results showed that the ability of BLK to change its own tyrosine kinase activity in response to intracellular ROS changes, but it remains unclear exactly how BLK receives the molecular signal of ROS level changes in the cells and how it converts these signals into its own tyrosine kinase activity to activate phosphorylation signals. According to our PredictProtein results, the amino acids at positions 313, 367, 368, 450, 454, and 466 in the kinase domain of BLK are cysteine residues. Previous studies have shown that cysteine residues are susceptible to covalent modification by oxidation and that the covalent modification of BLK is a necessary step for it to activate its own tyrosine kinase activity (*40,41*). Whether BLK receives signals indicating that ROS changes under oxidative stress through these cysteine residues in its kinase domain, before being covalently modified by ROS oxidation to activate its tyrosine kinase activity, remains to be explored in further studies. However, from a treatment perspective, the newly discovered molecular target of BLK is beneficial for improving the survival and efficacy of transplanted BMSCs.

Although this study has certain shortcomings, it was nevertheless able to discover which BMSC subgroup is resistant to apoptosis induced by oxidative stress, and it revealed a new role of BLK in enabling cells to acquire this type of resistance. Specifically, BLK can synergistically regulate redox and apoptotic signaling networks—including ERK, Akt, STAT, and NF-κB—to make BMSCs more resistant to oxidative stress and thus less susceptible to stress-induced apoptosis. Our findings therefore provide new strategies and regulatory targets for improving the survival and efficacy of transplanted BMSCs under oxidative stress

## Materials and Methods

### Clinical sample

Human experiments were performed in accordance with the Medical Science Ethics Committee of the Affiliated Hospital of Guizhou Medical University, and the experimental protocol was reviewed and approved by the Medical Science Ethics Committee of the Affiliated Hospital of Guizhou Medical University (2023 LUN Review No. 206). To acquire bone marrow samples, patients who were undergoing amputation at the Affiliated Hospital of Guizhou Medical University were voluntarily recruited, under a protocol that was approved by the institution’s ethics review board. The bone marrow was collected from severed limbs, after which the samples were diluted into a single-cell suspension so that the BMSCs could be isolated and cultivated.

### Animal experiments

Animals were provided by the experimental animal center of Guizhou Medical University, and the experimental protocol was approved by the Ethics Committee of Guizhou Medical University (No. 2200603). All procedures were accordance with the guidelines for the Care and Use of Experimental Animals issued by the National Institutes of Health (No. 85-23, revised 1996).

### Culture and identification of BMSCs

#### Isolation and culture of human BMSCs

The human bone marrow samples were mixed with phosphate-buffered saline (PBS) buffer at a 1:1 ratio, and the cell suspension was centrifuged for 10 min at 1,500 revolutions per minute (rpm) to obtain a cellular precipitate. The mononuclear cells were isolated from this precipitate using a Ficoll separation solution (1.077 g/ml). After cleaning the isolated mononuclear cells with 10 ml of PBS buffer, the cell suspension was centrifuged at 1,500 rpm for 10 min to obtain the mononuclear cell precipitate. Finally, the cell precipitates were suspended in 5 ml of Dulbecco’s modified Eagle's medium (DMEM) containing 10% fetal bovine serum (FBS) and 1% penicillin–streptomycin and then cultured at 37 °C and 5% CO_2_.

#### Isolation and culture of rat BMSCs

Femur and tibia tissues were extracted from young Sprague‒Dawley (SD) rats (male and female) weighing 25 g. The bone marrow cavities were washed with complete DMEM to obtain a mixture containing the bone marrow. The cell suspension was then centrifuged at 1,000 rpm for 5 min to obtain a cell precipitate. Finally, the cells were resuspended in 5 ml of DMEM, inoculated in a cell culture flask, and cultured at 37 °C and 5% CO_2_.

#### BMSC identification

Second-generation human BMSCs and rat BMSCs were selected for the induction of differentiation using osteogenic, chondrogenic, and lipogenic differentiation kits to identify their multidirectional differentiation competencies. The expression levels of CD73, CD90, CD105, CD45, CD34, and CD11b were detected by flow cytometry to identify cell surface antigen markers.

#### Isolation and culture of OSR-BMSCs

To obtain the subgroup of BMSCs with resistance to oxidative stress, we labeled BMSCs with CD90 and CD72 and then performed flow sorting. The detailed steps of this procedure were as follows: 50 μl of cell suspension, 10 μl of anti-CD90/fluorescein isothiocyanate (FITC) surface antigen antibody (BD, USA), and 10 μl of anti-CD72/phycoerythrin (PE) surface antigen antibody (BD) were mixed and incubated for 30 min. The cells were suspended with 500 μl of PBS buffer (containing 2% FBS), centrifuged at 800 rpm for 5 min, and then washed twice. Next, the cells were resuspended in 800 μl of PBS buffer containing 2% FBS. A collection tube containing l-DMEM and 20% FBS was prepared, and CD90^+^ cells were collected using a flow sorter (Beckman, USA). The CD90^+^ cells were then resuspended in 800 μl of PBS buffer containing 2% FBS, and any CD72^+^ cells were collected again using a flow sorter. Finally, the CD90^+^/CD72^+^ cells were resuspended in 5 ml of l-DMEM, inoculated in a cell culture flask, and cultured at 37 °C and 5% CO_2_.

### Oxidative stress model

In previous studies, we used H_2_O_2_ to simulate the oxidative stress microenvironment and successfully constructed the oxidative stress model [17,19]. In brief, when the fusion degree of the cultured cells reached 80 to 85%, the cells were treated with DMEM complete medium with 1,000 μM H_2_O_2_ at 37 °C and 5% CO_2_ for 24 h.

### Identification of resistance to oxidative stress in BMSCs

The BMSCs were treated with DMEM containing different concentrations of H_2_O_2_ for 24 h. Their activity levels were then detected using a cell counting kit-8 (CCK-8), and their apoptosis rate was determined using flow cytometry. A fitting curve was drawn in GraphPad Prism 8.0 software to evaluate the effects of H_2_O_2_ on BMSC activity and apoptosis. The IC_50_ value was defined as the concentration of H_2_O_2_ at which the cell viability dropped to 50%, or the cell apoptosis rate reached 50%. The RI of the BMSCs was calculated based on the IC_50_ value, as follows: RI = IC_50_ (BMSCs derived from bone graft area)/IC_50_ (BMSCs derived from bone marrow). An RI > 5 indicated BMSCs with resistance to oxidative stress.

### Proteomic and phosphorylated proteome analysis

The cell precipitation samples were divided into 2 groups: sensitive and resistant. Each group had 3 duplicate biological samples. The total cell protein was extracted via SDT Lysis Buffer and quantified using the bicinchoninic acid assay (BCA) method. The primary structure of the proteins was exposed to dithiothreitol and iodoacetamide, after which the protein suspension was digested with 2 μg of trypsin at 37 °C overnight to collect the peptide filtrate. The peptides were desalted and purified using Sep-Pak tC18 cartridges, and the peptide content was detected using an enzymolysis assay [59].

Phosphorylated peptide enrichment: After each sample was purified by desalting, an equal amount of peptide was taken and enriched in series in accordance with the instructions in the High-Select Fe-NTA phosphopeptide enrichment kit. The collected TiO_2_ eluents were dried in a vacuum centrifuge concentrator.

The lyophilized elution products (total protein peptides or phosphorylated protein peptides) were redissolved in 0.1% formic acid (FA) aqueous solution and separated using NanoElute chromatography. After chromatographic separation, the samples were analyzed using the PASEF mode of a timsTOF Pro mass spectrometer. Mass spectrometry (MS) data were analyzed using MaxQuant software version 1.6.17.0, and MS data were searched in the UniProt rat proteome database. Protein abundance was calculated using normalized protein spectral intensity, and proteins with difference multiples > 2 or < 0.5 and *P* values < 0.05 were considered differentially expressed proteins or differentially phosphorylated peptides [60–62].

### Bioinformatics analysis

First, the quantitative information of the differentially expressed target protein sets was normalized, after which the 2 dimensions of sample and protein expression were hierarchically clustered using cluster analysis software to generate hierarchical clustering heat maps. Blast2Go software (https://www.blast2go.com/) was used to annotate the Gene Ontology (GO) function of all differentially expressed proteins and count them [63,64]. KEGG or GSEA was performed on the differentially expressed target protein sets by comparing the distributions of the target protein and total protein sets within the signaling pathways. The protein belonging to the phosphorylation site with the most significant expression (*P* < 0.05) was selected as the target protein for direct interaction network analysis. The interaction between the proteins was analyzed in the STRING database using the target protein ID, and the interaction network visualization results were generated using Cytoscape software.

### Lentivirus transfection

BLK gene-overexpression and BLK gene-interference lentiviruses were purchased from GeneChem (Shanghai, China). Techniques such as overexpression and interference lentivirus transfection and screening of the positive cells have been established and described in previous studies [19,20]. In addition, a lentivirus with GFP tag was constructed, which can successfully express GFP after transfection, and could be used to label transplanted BMSCs. All BMSCs that were modified via gene transfection were established using the same strategy. Briefly, the BMSCs were inoculated in 6-well plates, and the optimal multiplicity of infection (MOI) of the lentivirus was identified in a pre-experiment. The lentivirus and Hitrans P were then added to infect the BMSCs at 37 °C and 5% CO_2_ for 12 h. After 5 d of lentivirus infection, any positive cells that were successfully infected with lentivirus were obtained via continuous screening with puromycin. RNA was extracted from these isolated cells to detect gene expression.

### Real-time qPCR

Total RNA was extracted by TRIzol, and the RNA concentration and purity were detected by Nanodrop 2000 nucleic acid quantifier. The Oligo (dT) primer and M-MuLV reverse transcriptase were used as the reaction system to synthesize cDNA. Then, the cDNA was used as the template for PCR amplification on the real-time fluorescence quantitative PCR iCycler iQ instrument, and the 2^−∆∆Ct^ method was used to analyze the results of real-time qPCR to calculate the relative expression of RNA.

### Western blotting

The protein was extracted by cell lysis buffer (radioimmunoprecipitation assay) and quantified by BCA protein concentration detection kit. An equal amount of denatured protein samples was added to sodium dodecyl sulfate–polyacrylamide gel electrophoresis (SDS-PAGE) gel tank for electrophoresis. After electrophoresis, the protein was imprinted on methanol-activated polyvinylidene fluoride (PVDF). Subsequently, the blots were blocked with 5% skim milk powder solution at room temperature for 1 h. Primary antibodies, including BLK, p-BLK, p-Bad, p-CASP9, Survivin, IAP1/2, Bfl-1 (Cell Signaling Technology), Bcl-2, Bcl-XL, c-Myc, CAT, MnSOD, HO-1, GR, ERK1/2, p-ERK1/2, Akt, p-Akt, STAT3, p-STAT3, NF-κB, p-NF-κB, and β-actin (Abcam), were incubated at 4 °C overnight. Next, the horseradish peroxidase (HRP)-immunoglobulin G (IgG) secondary antibody was incubated at room temperature for 1 h. After the PVDF membrane was washed with tris-buffered saline with tween-20‌ (TBST), the gel imaging system was used to expose and collect pictures.

### ROS level detection

The detection solution was prepared in accordance with the instructions of the fluorescence probe-dihydroethidium (DHE) detection kit, and then 400 μl of the detection solution was added into the cell culture dish and incubated at 37 °C for 30 min. Finally, after the cells were washed with PBS, they were covered with anti-fluorescence quencher, and the fluorescence intensity was analyzed by confocal laser microscopy.

### Flow cytometry assay

The cells were digested with trypsin to obtain cell precipitates. The cell precipitates were washed twice with PBS, and 5 μl of Annexin V-FITC and 5 μl of propidium iodide (PI) were added for cell labeling. The cells were incubated at room temperature for 15 min, and finally 500 μl of staining buffer was added to each tube, and the apoptosis rate was detected by flow cytometry.

### TUNEL staining

The cells were fixed with paraformaldehyde for 30 min, and the immunostaining solution permeated the cells for 5 min. The cells were labeled with TUNEL detection solution for 90 min, and 4′,6-diamidino-2-phenylindole (DAPI) for 5 min. Finally, the cells were covered with anti-fluorescence quencher, and the TUNEL^+^ cells were detected under a confocal laser microscope.

### Immunofluorescence detection of nuclear translocation

The cells were fixed with paraformaldehyde for 10 min and sealed with immunostaining sealer for 1 h. The primary antibody reaction was then performed with ERK1/2, Nrf2, STAT3, and NF-κB (Abcam). The cells were incubated at 4 °C overnight, followed by another incubation with Cy3-labeled fluorescent secondary antibody for 1 h. The nuclei were labeled with DAPI. Finally, the cells were covered with anti-fluorescence quencher, and any red fluorescence was observed using a confocal laser microscope.

### Tissue-engineered bone construction and cell biocompatibility detection

XACB was acquired from Shanghai Yapeng Biological Technology Co. Ltd. A cell suspension with a density of 2 × 10^7^ cells/ml was slowly dripped into XACB and buffered in an incubator for 3 h. Complete l-DMEM was added to cover the XACB and cultured in the incubator to construct the tissue-engineered bone. Five pieces of tissue-engineered bone were extracted every day for detection via CCK-8, and cell growth curves were plotted. On day 6, the growth of the cells on the XACB was observed via scanning electron microscopy to determine the biocompatibility of the BMSCs with XACB.

### The model of SONFH

The model of SONFH was established as previously described [65]. Briefly, the adult male SD rats were injected intravenously with lipopolysaccharides (2 mg/kg) for 2 d, followed by methylprednisolone (60 mg/kg) into the gluteal muscle for 7 d. At week 8, the magnetic resonance imaging (MRI) was used to evaluate necrosis in the femoral head.

### Magnetic resonance imaging

The SD rats were anesthetized with 0.3% pentobarbital sodium and scanned using a 7.0 T nuclear magnetic resonance (NMR) apparatus with the following parameters: repetition time, 2,200 ms; echo time, 35 ms; scan time, 2,520 s; field of view, 64 mm × 64 mm; matrix size, 256 × 256; slice thickness, 1 mm. RadiAnt DICOM Viewer was used to process the image and observe the femoral head signal.

### Tissue-engineered bone transplantation

The SD rats were anesthetized with 0.3% pentobarbital sodium. We opened their hips and the femoral heads, layer by layer, in a prone position. Based on the preoperative MRI results, the necrotic area was located using a 0.5-mm micro-drill-bit, and the necrotic bone tissue was thoroughly scraped with a 2-mm spherical grinding drill. Each operative area was rinsed with normal saline, the tissue-engineered bone was implanted, and the pore was filled with bone wax.

### Live imaging of animals

The tissue-engineered bone was constructed using BMSCs that were labeled with DiR. At 0 and 48 h after the tissue-engineered bone transplantation, the rats were anesthetized with pentobarbital sodium (30 mg/kg), and the DiR fluorescence in the region of interest was collected and analyzed using a Spectrum in vivo imaging system (IVIS) with Living Image software version 4.4.

### Micro-computed tomography

The femoral head tissues of the SD rats were obtained after they were anesthetized. The femoral heads were fixed with paraformaldehyde, and the images were collected by micro-CT at 85 kVp and 200 μA with a resolution of 6.5 μm. NRecon 1.6 (Skyscan), CTAn 1.9 (Skyscan), and CTVol 2.0 (Skyscan) were used to analyze the parameters of the trabecular bone in the region of interest.

### Detection of the ROS of the femoral head

The SD rats were injected with DHE fluorescence reagent (20 mg/kg) at the graft area 24 h after tissue-engineered bone transplantation and then euthanized. Their femoral heads were removed, fixed with 4% paraformaldehyde, and decalcified with EDTA. They were then incubated with 2% polyvinylpyrrolidone and 20% sucrose solution at 4 °C for 24 h. The femoral head tissues were then frozen and sliced. DAPI was used to stain the nuclei. The ROS content in the transplantation area was analyzed using a confocal laser microscope.

### TUNEL staining of bone tissue

The femoral head tissue was cut into 5-μm-thick sections after being treated via paraformaldehyde fixation, EDTA decalcification, ethanol dehydration, and paraffin embedding. After dewaxing, the sections were incubated with TdT incubation buffer at 37 °C for 2 h and the nuclei were labeled with DAPI. Finally, the sections were sealed with neutral balsam and observed via fluorescence microscopy.

### Immunofluorescence assay

The femoral head specimens were fixed, decalcified, embedded, sliced, and dewaxed. Bone tissue antigen repair solution was used for antigen repair. A diluted solution of primary antibody (CAT monoclonal antibody, Bcl-2 monoclonal antibody, Runx2 monoclonal antibody) was incubated at 4 °C overnight. Next, they were incubated with secondary antibody at 37 °C for 1 h in the dark. The sections were then sealed with neutral balsam and observed via fluorescence microscopy.

### H&E and Masson staining

The femoral head tissue of SD rats was obtained after anesthesia. The bone tissue was cut into 4-μm-thick sections after being treated with paraformaldehyde fixation, EDTA decalcification, ethanol dehydration, and paraffin embedding, respectively. The sections were stained with H&E and Masson according to kit instructions. Finally, the sections were observed under the microscope.

### Statistical analysis

The experimental data were analyzed using SPSS 20.0 and GraphPad Prism 8.0 (USA). The data of normal distribution were described by the mean ± SD, and statistical significance was determined by 2-tailed unpaired Student’s *t* tests (2 groups) and Tukey’s post hoc test after one-way analysis of variance (ANOVA) (≥3 groups). The data of non-normal distribution were described by the median (P25, P75) values, and the statistical significance between groups was tested by Kruskal–Wallis rank-sum test. *P* < 0.05 was considered statistically significant.

## Data Availability

All data are available in the main text or the Supplementary Materials.
