# Supplementary figures and images for: B-Lymphoid Tyrosine Kinase Crosslinks Redox and Apoptosis Signaling Networks to Promote the Survival of Transplanted Bone Marrow Mesenchymal Stem Cells
Source: Research (Wash D C). 2025 Apr 15;8:0660. doi: 10.34133/research.0660 (PMC11999575; doi:10.34133/research.0660)

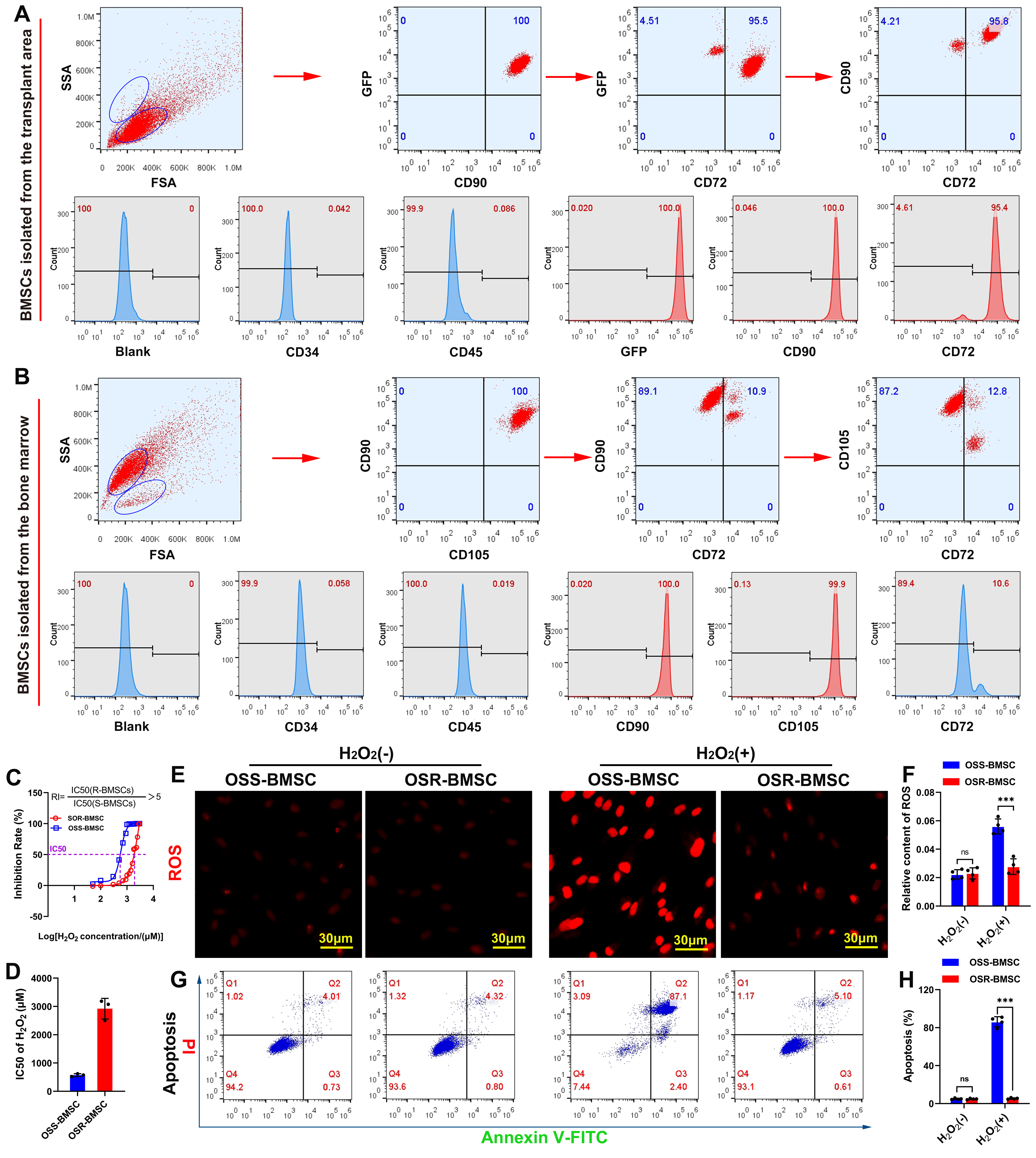

Supplement: Supplementary 1 — Figs. S1 to S6 [file research.0660.f1.zip › Supplementary figure 2.jpg]

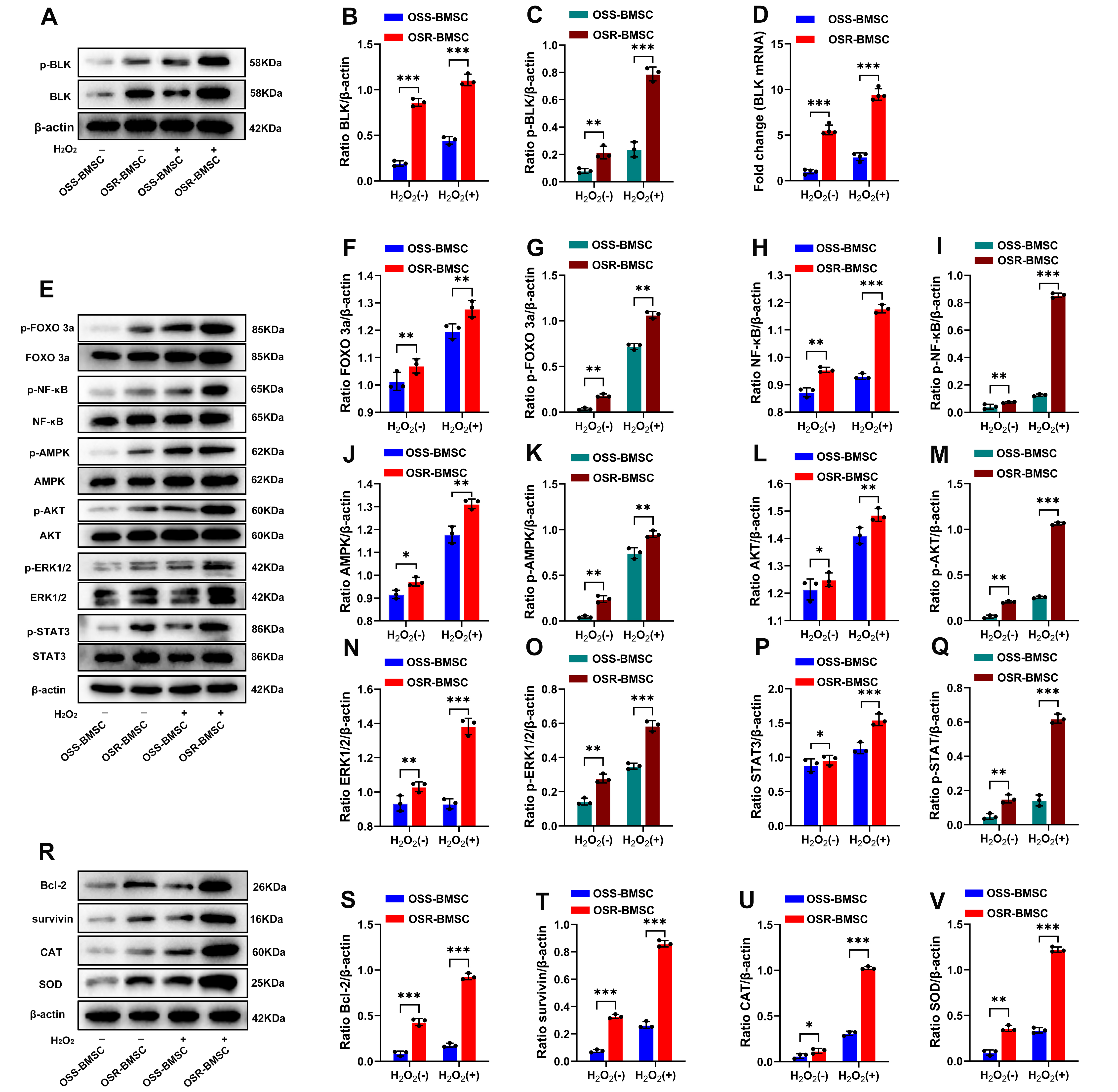

Supplement: Supplementary 1 — Figs. S1 to S6 [file research.0660.f1.zip › Supplementary figure 3.jpg]

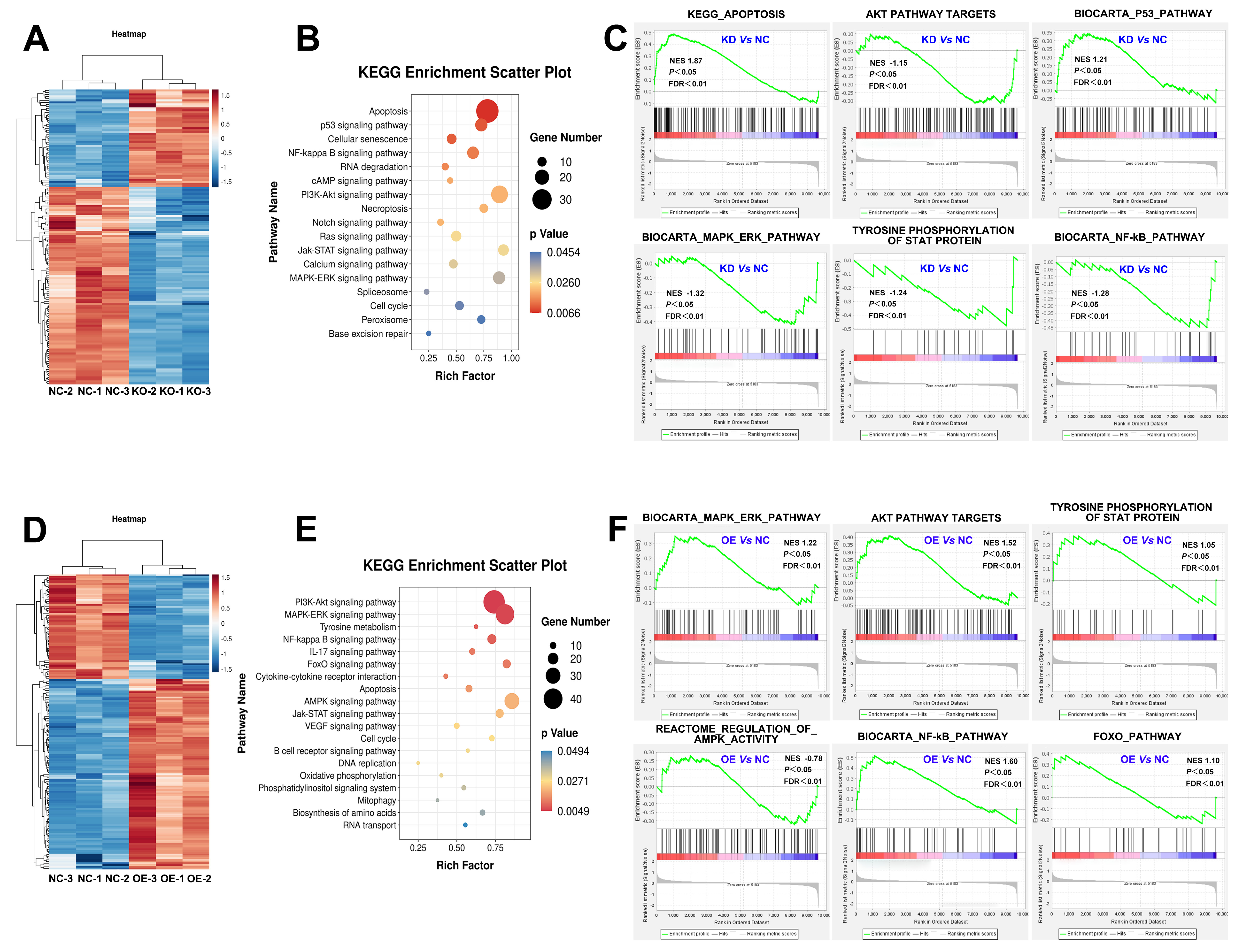

Supplement: Supplementary 1 — Figs. S1 to S6 [file research.0660.f1.zip › Supplementary figure 6.jpg]
